# Identification of single nucleotide polymorphisms (SNPs) associated with chronic graft-versus-host disease in patients undergoing allogeneic hematopoietic cell transplantation

**DOI:** 10.1007/s00520-023-08044-3

**Published:** 2023-09-21

**Authors:** Jean-Luc C. Mougeot, Micaela F. Beckman, Allan J. Hovan, Bengt Hasséus, Karin Garming Legert, Jan-Erik Johansson, Inger von Bültzingslöwen, Michael T. Brennan, Farah Bahrani Mougeot

**Affiliations:** 1 Translational Research Laboratories, Department of Oral Medicine/Oral & Maxillofacial Surgery, Atrium Health - Carolinas Medical Center, Charlotte, NC USA; 2 BC Cancer, Oral Oncology and Dentistry, Vancouver, BC Canada; 3 Department of Oral Medicine and Pathology, University of Gothenburg, Gothenburg, Sweden; 4 Karolinska Institutet, Huddinge, Sweden; 5 Department of Hematology and Coagulation, Sahlgrenska University Hospital, Gothenburg, Sweden; 6 Department of Oral Microbiology and Immunology, University of Gothenburg, Gothenburg, Sweden; 7 Department of Otolaryngology/Head & Neck Surgery, Wake Forest University School of Medicine, Winston-Salem, NC USA

**Keywords:** Chronic graft-versus-host disease, Allogeneic hematopoietic cell transplant, Whole exome sequencing, Single nucleotide polymorphism, Multi-marker gene-level analysis, JAK pathway

## Abstract

**Introduction:**

Chronic graft-versus-host disease (cGVHD) is a debilitating side effect of allogeneic hematopoietic cell transplantation (HCT), affecting the quality of life of patients. We used whole exome sequencing to identify candidate SNPs and complete a multi-marker gene-level analysis using a cohort of cGVHD( +) (*N* = 16) and cGVHD( −) (*N* = 66) HCT patients.

**Methods:**

Saliva samples were collected from HCT patients (*N* = 82) pre-conditioning in a multi-center study from March 2011 to May 2018. Exome sequencing was performed and FASTQ files were processed for sequence alignments. Significant SNPs were identified by logistic regression using PLINK2_v3.7_ and Fisher’s exact test. One cGVHD( −) patient sample was excluded from further analysis since no SNP was present in at least 10% of the sample population. The FUMA platform’s SNP2GENE was utilized to annotate SNPs and generate a MAGMA output. Chromatin state visualization of lead SNPs was completed using Epilogos tool. FUMA’s GENE2FUNC was used to obtain gene function and tissue expression from lead genomic loci.

**Results:**

Logistic regression classified 986 SNPs associated with cGVHD( +). SNP2GENE returned three genomic risk loci, four lead SNPs, 48 candidate SNPs, seven candidate GWAS tagged SNPs, and four mapped genes. Fisher’s exact test identified significant homozygous genotypes of four lead SNPs (*p* < 0.05). GENE2FUNC analysis of multi-marker SNP sets identified one positional gene set including lead SNPs for *KANK1* and *KDM4C* and two curated gene sets including lead SNPs for *PTPRD*, *KDM4C*, and/or *KANK1*.

**Conclusions:**

Our data suggest that SNPs in three genes located on chromosome 9 confer genetic susceptibility to cGVHD in HCT patients. These genes modulate *STAT3* expression and phosphorylation in cancer pathogenesis. The findings may have implications in the modulation of pathways currently targeted by JAK inhibitors in cGVHD clinical trials.

**Supplementary information:**

The online version contains supplementary material available at 10.1007/s00520-023-08044-3.

## Introduction

Each year thousands of patients suffering from blood disorders, hematological cancers, or immunodeficiency complete conditioning therapy prior to undergoing allogeneic hematopoietic stem cell transplant (HCT) in the USA [1]. In allogeneic HCT, hematopoietic cells are isolated from a related or unrelated donor and must have the same human leukocyte antigen (HLA) types as the recipient to be considered “HLA matched.” The types of HLAs typically checked are HLA-A, B, C, DRB1, and DPB1 haplotypes on chromosome 6 that are inherited [2, 3]. If no related donors with matched or partial matched HLA types are available, unrelated donors with full or partial HLA matches are preferred [3]. Those HCT patients with HLA mismatched donors (i.e., having one or more HLA type that is not identical) experience increased rates of chronic graft-versus host disease (cGVHD) and even with identical HLA matches, up to 41% of patients undergoing HCT develop cGVHD [1, 4]. When donor T cells respond to host cells, GVHD can occur [5]. cGVHD development process consists of three phases: (1) afferent phase (2) donor T cell activation, differentiation, and migration and (3) effector phase [6]. If occurring 100 days post-transplantation, GVHD is considered chronic as opposed to acute [7]. cGVHD can cause significant debilitating symptoms. Patients with cGVHD experience a lower quality of life with daily activities being impaired long after clinical symptoms of cGVHD appear. Many tissues can be affected by cGVHD including the eyes, gastrointestinal (GI) tract, liver, lungs, skin, and mouth [8].

Symptoms of cGVHD are scored on a grading scale of zero to three with zero corresponding to no organ involvement and three corresponding to the medical condition in which the function of the organ is severely compromised [9]. Furthermore, global severity of cGVHD can be described as mild, moderate, or severe. Mild cGVHD is characterized by a patient having one to two organs affected with a score of one. Moderate cGVHD is described as three or more organs affected with a score of one, at least one organ with a score of two, and a lung score of one, while severe cGVHD has at least one organ with a score of three and a lung score of two [9].

Clinical treatment of cGVHD aims to improve and to stabilize organ involvement and symptoms, but also to improve quality of life and survival [10]. Guidelines recommend no treatment or local application of steroids or other immunosuppressive drugs if mild cGVHD [11]. With moderate or severe cGVHD, the first treatment is systemic steroids. If refractory to steroids, first choice is ruxolitinib and second choice other immunosuppressive treatments such as ECP, ibrutinib, rituximab, or sirolimus [12]. The reason why some HCT patients develop more severe cGVHD is not well understood, although some studies have sought to associate genetic polymorphisms with cGVHD [13–16]. Previous studies have associated SNPs mapped to genes with cGVHD incidence including C-X-C motif chemokine receptor 3 (*CXCR3*), C–C motif chemokine receptor 6 (*CCR6*), FGFR1 oncogene partner (*FGFR10P*), interleukin 10 (*IL10*), interleukin 1 receptor type 1 (*IL1R1*), nuclear factor kappa B subunit 1 (*NFKB1*), heparanase (*HSPE*), and cytotoxic T-lymphocyte associated protein 4 (*CTLA4*) [13, 15–17].

Here, we carried out whole exome sequencing to (i) identify candidate single nucleotide polymorphisms (SNPs) associated with cGVHD and (ii) complete a multi-marker gene-level analysis, using a cohort of 82 allogeneic HCT patients, to better understand the underlying genetic susceptibilities distinguishing patients who developed cGVHD (*N* = 16) from those who did not (*N* = 66).

## Methods

### Chronic graft-versus-host disease determination

Participants were enrolled in a multicenter prospective study (OraStem) assessing oral complications related to conditioning regimens, hematopoietic cell transplantation, and immunologic reactions (mainly cGVHD) that may follow and as previously described [18]. Enrollment started at the first center in March 2011 and proceeded at intervals until May 2018. Hematological conditions treated included blood cancers or blood disorders, namely, myeloma, lymphoma, acute/chronic lymphoblastic leukemia (ALL/CLL), acute/chronic myelogenous leukemia (AML/CLL), myelodysplastic syndrome, myelofibrosis, or immune deficiency. Conditioning regimens included either non-myeloablative, myeloablative, or reduced intensity conditioning. Participants were assessed prior to the start of HCT and 100 days, 6 months, and 1 year after HCT. cGVHD was assessed prospectively on site at each follow-up visit as part of Orastem. A specific question regarding oral cGVHD was also assessed. A participant was considered positive if they were noted by the oncologist to have cGVHD at 100 days, 6 months, or 1 year after HCT. Data was not collected on grade or organ involvement of cGVHD. This study was approved by Atrium Health IRB# IRB00080071. All participants have signed an informed consent form.

### Saliva sample collection, DNA isolation, library prep, and sequencing

Whole saliva was collected from adult patients from three international sites (Atrium Health Carolinas Medical Center, Charlotte, NC, USA; BC Cancer, Vancouver, BC, Canada; and two Swedish sites-Sahlgrenska University Hospital, Gothenburg and Karolinska Institute, Huddinge, Sweden) at baseline prior to conditioning therapy into Oragene collection tubes (DNA GenoTek, Ottawa, Ontario, Canada). Samples from the satellite sites were stored in the Oragene kit and were shipped at ambient temperatures to the Translational Research Laboratory at Atrium Health Carolinas Medical Center (AH-CMC), Charlotte, NC, USA. All samples were processed per manufacturer’s instructions and stored at − 80 °C until further analysis. Isolation of DNA was completed using the prepIT-L2P reagent (DNA GenoTek, Ottawa, Ontario, Canada), following manufacturer’s instructions (DNA GenoTek, Inc.).

Following DNA extraction and QC profiling, the samples were submitted for library preparation and sequencing. Libraries were generated for each sample using NEB reagents for PCR construction and PFX polymerase for the post ligation PCR. KAPA RT-PCR was completed on these libraries before hybridization. NimbleGen_v3.0_ hybridization reactions were completed, and resulting libraries underwent final library QC analysis which included KAPA qPCR. A Paired-end100 sequencing run was completed via the Illumina Hiseq platform.

### Processing sequencing reads for SNP determination and bioinformatic analysis

Quality control was completed using FASTQC_v0.11.9_ on the paired-end FASTQ files of 16 cGVHD( +) and 66 cGVHD( −) samples [19]. Adapters and low-quality reads were trimmed and paired ends were combined and aligned to the GRCh38.19 reference genome [20–22]. Duplicates were marked, reads were aligned, and base quality score recalibration was completed using GATK_v4.2.6.1_ tools [23].

Variants were called using GATK_v4.2.6.1_’s HaplotypeCaller [23]. Variant Call Files (VCF) were combined using BCFtools_v1.9_ and filtered using VCFtools_v0.1.16_ keeping only SNPs present in > 10% of the population with a minor allele frequency > 1% and removing multi-allelic SNPs [24, 25]. One cGVHD − patient sample was removed from downstream analysis due to not having any SNPs present in at least 10% of the sample population. Variant association analysis was then completed on filtered SNPs using PLINK2_v3.7_ by performing a logistic regression analysis on cGVHD + (*n* = 16) and cGVHD − SNPs (*n* = 65 of 66 samples) [26]. The genomic inflation factor of our dataset was < 1.0; therefore, covariates were not included in the logistic regression. A principal component analysis (PCA) plot of the data was created using Python_v3.9.13_ programming language packages [27–29].

Significant SNP positions were loaded into the Known VARiants (Kaviar_v160204_) online tool with GRCh38.19 used as the reference, and Reference SNP cluster IDS (RSids) were retrieved [30]. The Functional Mapping and Annotation of Genome-Wide Association Studies (FUMA) online platform was used to annotate SNPs with the SNP2GENE tool and Multi-marker Analysis of GenoMic Annotation (MAGMA) was utilized [31]. Options included the cGVHD association logistic regression results from PLINK2_v3.7_ as the input, significant SNPs with RSids as the “lead snp” list (*n* = 909 unique out of 986 total SNPs), sample size as “*N* = 81,” a window of 2.5kbp upstream and 0 kbp downstream and a maximum adjusted *p*-value of 1 × 10^−5^. Manhattan plots showing the GWAS summary statistics (*p* < 1 × 10^−5^) and the gene-based test (*p* < 6.219 × 10^−5^) were created. A contingency table of observed heterozygous vs. homozygous minor alleles was then produced for cGVHD − and cGVHD + groups, and a post hoc Fisher’s exact test was used for the lead SNPs using R_v4.2.1_. Chromatin state genomic visualization maps of the lead candidate SNPs were created using the Epilogos online tool with “hg38” as the human reference genome and “All 127 Roadmap Epigenomes” in the 18-state model [32].

The FUMA GENE2FUNC online tool was used to obtain gene function and tissue expression (GTEx_v8_ 54 and 30 tissue types) from SNP2GENE identified lead genomic loci (i.e., lead SNPs). Additionally, GENE2FUNC was run on all significant SNPs annotated to genes (*n* = 804) with recognized Ensembl IDs (*n* = 679; 678 unique) and all known background genes within the online tool (*n* = 57,241; 35,142 unique), using hypergeometric tests to determine overrepresented genes of interest (padj < 0.05).

## Results

**Fig. 1:**
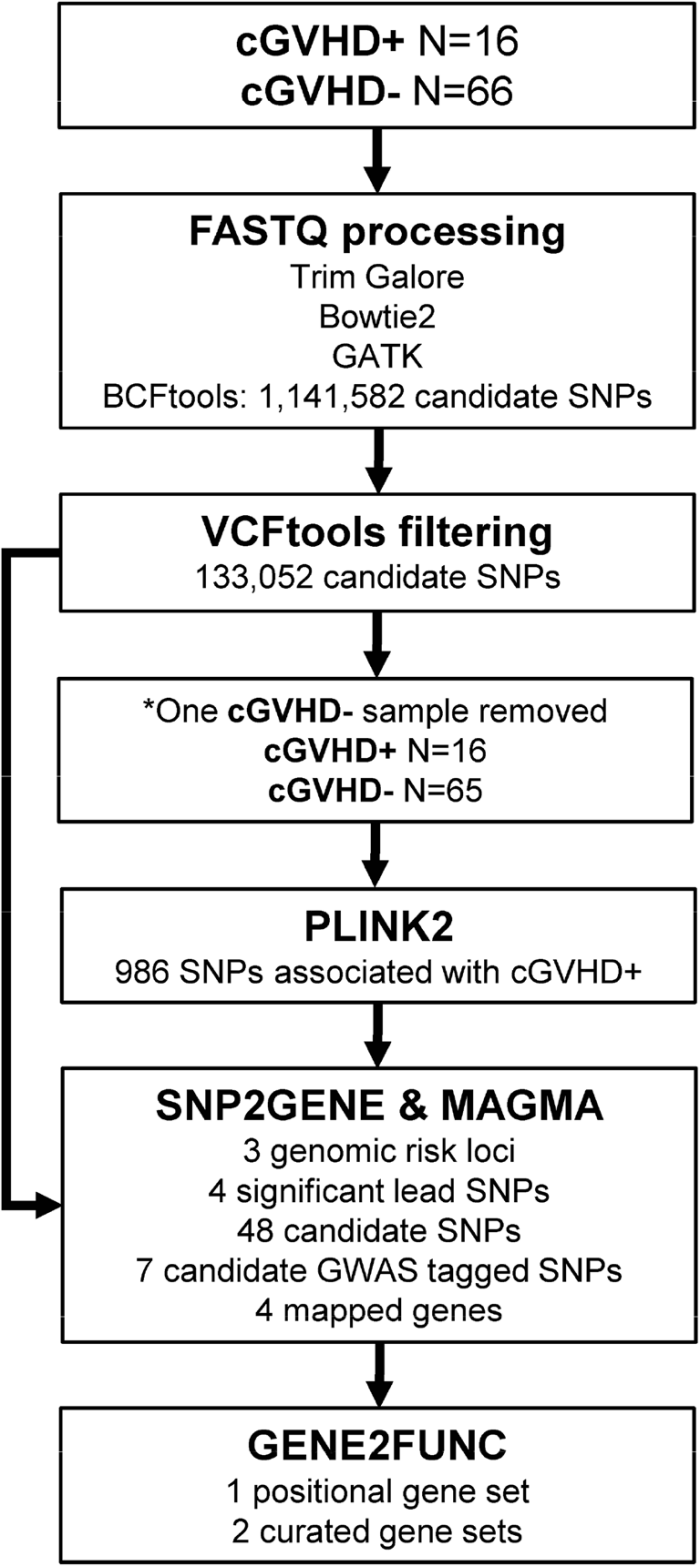
Analytical design for determination of SNPs associated with cGVHD in a cohort of HCT patients by exome sequencing. Legend: Samples were collected from 82 hematopoietic cell transplant (HCT) recipients either with chronic graft-versus-host disease (cGVHD + ; *N* = 16) or without (cGVHD − ; *N* = 66). FASTQ processing was completed using Trim Galore_v0.6.7_, Bowtie2_v2.4.1_, Genome Analysis Toolkit (GATK_v4.2.6.1_), and BCFtools_v1.9_. VCFtools_v0.1.16_ was used to filter 1,141,582 identified single nucleotide polymorphisms (SNPs) keeping only SNPs present in > 10% of the population, a minor allele frequency of > 1%, and removing multi-allelic SNPs. Filtering resulting in 133,052 candidate SNPs for logistic regression analysis in PLINK2_v3.7_. PLINK2_v3.7_ classified 986 SNPs to be significantly associated with cGVHD + . Using the 133,052 SNPs with the 986 significant SNPs as SNP2GENE and MAGMA input, significant returned results included three genomic risk loci, four lead SNPs, 48 candidate SNPs, seven candidate GWAS tagged SNPs, and four mapped genes. Using these outcomes as input for GENE2FUNC resulted in three significant functional gene sets including one positional and two curated ones. *One cGVHD − sample was removed by not having any SNPs present in at least 10% of the sample population

Patient demographics are presented in Table 1. Overall, analytical strategy with associated summary results is presented in Fig. 1. Filtering with VCFtools_v0.1.16_, resulted in 133,052 candidate SNPs being retained out of 1,141,608 total. One sample had to be removed from further analysis by not having any SNPs present in at least 10% of the sample population from the cGVHD( −) group resulting in 81 total patient samples used for analysis. Using PLINK2_v3.7_, 986 candidate SNPs from the 81 patient samples (cGVHD + , *n* = 16; cGVHD − , *n* = 65) were found significant as determined by logistic regression (Online Resource 1). A PCA plot showing the first four principal components of the cGVHD + and cGVHD − groups based on SNP data is presented in Fig. S1 (Online Resource 2). Limited clustering was observed for cGVHD − patients, while cGVHD + patients were scattered, likely due to differences in sample sizes. There were, however, no significant differences in the variation of SNPs in each group, indicating this result being independent from demographics.

**Table 1:** Demographics of patient cohort undergoing hematopoietic cell transplant

Criteria^a^	*cGVHD − ^b^	cGVHD + ^c^	Combined^d^
Patient (M/F)^e^	66 (43/23)	16 (9/7)	82 (52/30)
Hematological condition^f^			
Acute myelogenous leukemia (M/F)	20 (16/4)	7 (3/4)	27 (19/8)
Chronic myelogenous leukemia (M/F)	2 (1/1)	1 (1/0)	3 (2/1)
Acute lymphoblastic leukemia (M/F)	6 (3/3)	3 (1/2)	9 (4/5)
Chronic lymphoblastic leukemia (M/F)	1 (1/0)	2 (1/1)	3 (2/1)
Myeloma (M/F)	13 (6/7)	0 (0/0)	13 (6/7)
Lymphoma (M/F)	14 (9/5)	2 (2/0)	16 (11/5)
Blood disorders (M/F)	10 (7/3)	1 (1/0)	11 (8/3)
Age^g^			
Median	55	58	55
Mean	50.77	52.31	51.07
Standard deviation	12.88	11.39	12.62
Range	18–69	27–66	18–69

^**a**^Demographic data for a cohort of patients undergoing allogeneic hematopoietic cell transplant (HCT) who did or did not develop chronic graft-versus-host disease (cGVHD)

^**b**^Group defined as graft-versus-host disease negative (cGVHD −)

^**c**^Group defined as graft-versus-host disease positive (cGVHD +)

^**d**^cGVHD − and cGVHD + groups combined

^**e**^Patient count and gender of patient: M is male, and F is female

^**f**^Hematological condition treated: M is male, and F is female. Blood disorders included myelodysplastic syndrome, myelofibrosis, immune deficiency, or other

^**g**^Median, mean, standard deviation, and range of patients’ age

Note: Due to the samples being collected from international satellite sites, ethnicity data was not collected. *One female cGVHD − myeloma patient sample was removed from further analysis due to the absence of SNPs present in at least 10% of the sample population

Using MAGMA output in the SNP2GENE analysis, we identified three significant genomic risk loci, four lead candidate SNPs, and 48 candidate SNPs to be significantly correlated with the development of cGVHD from a possible 909 annotated SNPs among the 986 identified by logistic regression. Manhattan plots at the SNP level and gene-level analyses are presented in Fig. S2 (Online Resource 3). The four lead candidate SNPs rs7031287, rs2296055, rs7030531, and rs10405821 mapped to the genes protein tyrosine phosphatase receptor type D (*PTPRD*), KN motif and ankyrin repeat domains 1 (*KANK1*), lysine demethylase 4C (*KDM4C*), and paralemmin (*PALM*), respectively. The four lead SNPs and 48 candidate SNPs are presented in Table 2. No lead SNPs had combined annotation dependent depletion (CADD) scores greater than suggested threshold of 12.37 [33]. However, CADD scores of two candidate SNPs (rs10975820 [*KANK1*:*R130C19.3*] and rs4465021 [*KDM4C*]) not identified as lead SNPs, exceeded this threshold (Table 2).

**Table 2:** SNP2GENE/MAGMA identified 48 candidate SNPs and four lead SNPs

SNP rsID^a^	Chr^b^	Pos^c^	Ref^d^	Alt^e^	Nearest gene^f^	CADD score^g^
rs7856734	9	631,439	A	C	KDM4C	1.922
rs9408651	9	655,962	G	T	KANK1	6.044
rs10739107	9	660,851	T	C	KANK1	1.236
rs2296055	**9**	**676,954**	**C**	**T**	**KANK1:R130C19.3**	**7.932**
rs4740851	9	677,986	A	T	KANK1:R130C19.3	7.468
rs10815466	9	680,714	G	A	KANK1:R130C19.3	5.244
rs73639400	9	683,423	C	T	KANK1:R130C19.3	5.124
rs12004436	9	683,430	C	T	KANK1:R130C19.3	2.111
rs10975820	9	684,160	A	G	KANK1:R130C19.3	12.55*
rs61562446	9	6,828,874	GA	G	KDM4C	1.009
rs56011297	9	6,830,379	C	T	KDM4C	2.992
rs113555302	9	6,831,369	T	TTTTA	KDM4C	0.898
rs62533755	9	6,832,134	C	T	KDM4C	1.376
rs7875118	9	6,832,456	C	T	KDM4C	0.602
rs7030531	**9**	**6,835,419**	**C**	**T**	**KDM4C:R130C19.3**	**10.86**
rs4467991	9	6,840,584	A	G	KDM4C	3.382
rs4465021	9	6,847,072	C	T	KDM4C	12.63*
rs10815471	9	6,848,369	C	G	KDM4C	1.114
rs10975853	9	6,848,524	C	G	KDM4C	1.271
rs56172510	9	6,855,391	A	G	KDM4C	2.471
rs12006340	9	6,856,646	A	C	KDM4C	1.662
rs1323588	9	8,453,781	C	T	PTPRD	4.222
rs10739165	9	8,454,265	G	T	PTPRD	8.644
rs7031287	**9**	**8,454,566**	**C**	**T**	**PTPRD**	**10.18**
rs7872683	9	8,455,012	A	G	PTPRD	5.565
rs7873571	9	8,455,031	A	C	PTPRD	4.149
rs6510920	19	728,469	A	G	PALM	3.609
rs7258499	19	728,476	G	T	PALM	1.658
rs4919887	19	728,626	C	T	PALM	0.087
rs4919822	19	728,628	C	T	PALM	0.237
rs4919823	19	728,799	C	T	PALM	0.998
rs4919888	19	728,983	A	G	PALM	1.402
rs4919824	19	729,049	C	T	PALM	0.664
rs75791909	19	729,180	A	AG	PALM	0.454
rs113501443	19	729,455	C	T	PALM	0.989
rs66565554	19	729,567	A	G	PALM	0.89
rs3787019	19	730,030	C	T	PALM	3.77
rs1009691	19	730,235	A	G	PALM	3.455
rs2067030	19	730,273	C	G	PALM	2.059
rs1009690	19	730,297	A	G	PALM	0.179
rs10405821	**19**	**731,055**	**C**	**G**	**PALM**	**1.834**
rs10405836	19	731,077	C	G	PALM	0.114
rs2074355	19	731,078	G	T	PALM	1.059
rs1050457	19	731,144	A	G	PALM	2.531
rs4483972	19	731,484	A	G	PALM	0.181
rs7257535	19	733,674	A	G	PALM	1.177
rs3761029	19	733,761	C	T	PALM	2.735
rs2283584	19	737,123	A	T	PALM	1.7

^**a**^Reference single nucleotide polymorphism (SNP) cluster ID (rsID) of candidate SNPs determined by the Functional Mapping and Annotation of Genome-Wide Association Studies (FUMA) SNP2GENE and Multi-marker Analysis of GenoMic Annotation (MAGMA) online tools with an input of all significant SNPs from PLINK2_v3.7_ (input = 909)

Significant lead SNPs (*p* ≤ 1 × 10^−5^) are shown in bold

^**b**^Chromosome of SNP

^**c**^Base pair location of SNP

^**d**^Reference allele

^**e**^Alternate allele

^**f**^Nearest gene symbol

^**g**^Combined Annotation Dependent Depletion (CADD) score of deleteriousness of predicted SNP using 63 functional annotations. SNPs with a CADD score greater than the suggested threshold of 12.37 are depicted using an asterisk (*)

The Fisher’s exact tests of the lead SNPs comparing the proportions of heterozygous and homozygous alternate genotypes resulted in three of the four lead SNPs being significant (*p* < 0.05) and one having a marginal significance (*p* = 0.0525) (Table 3 and Online Resource 4). We identified a total of 17 patients among 81, i.e., 14 in cGVHD − group and 3 in cGVHD + group with mutations in an intronic region of *PTPRD* (rs7031287; chr9:8,454,566: T > C). However, in the cGVHD( +) group, we identified three patient samples (18.75%) with homozygous mutations (C/C), while in the cGVHD( −) group, we observed 12 samples (18.18%) to be heterozygous and two samples (3.03%) to have a homozygous mutation (*p* < 0.05) (Table 3 and Online Resource 4). Genotyping of *KANK1* revealed heterozygous mutations in one sample (6.25%) of the cGVHD( +) group and in 11 samples (16.66%) of the cGVHD( −) group. Furthermore, we were able to identify homozygous alternate mutations in three (18.75%) of the 16 cGVHD( +) patients and in two (3.03%) cGVHD( −) samples. Using the Fisher’s Exact test, these proportions were determined as marginally significant (*p* = 0.0525). Genotyping of the SNP within *KDM4C* (rs7030531; chr9:6,835,419:C > T) revealed heterozygous mutations in none of the cGVHD( +) samples and in 14 samples (21.21%) of the cGVHD( −) group. Furthermore, we were able to identify homozygous alternate mutations in three (18.75%) cGVHD( +) patients and in none of the cGVHD( −) samples (*p* < 0.05) (Table 3 and Online Resource 4). The Fisher’s exact test on genotyping of the SNP within *PALM* (rs10405821; chr19:731,055:C > G) revealed that the proportions of heterozygous genotype vs. homozygous genotype in our cGVHD( +) and cGVHD( −) groups were significantly different (*p* = 5.85 × 10^−3^) (Table 3 and Online Resource 4).

**Table 3:** Fisher’s exact significance of SNP2GENE/MAGMA identified lead SNP genotypes

SNP^a^	Gene name^b^	Heterozygous^c^	Homozygous^d^	No allele detected^e^	Total # patients^f^	Fisher’s exact *p*-value^g^
rs7031287	**PTPRD**					
cGVHD +		0 (0%)	3 (18.75%)	13 (81.25%)	16	**0.0147**
cGVHD −		12 (18.46%)	2 (3.08%)	51 (78.46%)	65	
rs2296055	**KANK1**					
cGVHD +		1 (6.25%)	3 (18.75%)	12 (75%)	16	0.0525
cGVHD −		11 (16.92%)	2 (3.08%)	52 (80%)	65	
rs7030531	**KDM4C**					
cGVHD +		0 (0%)	3 (18.75%)	13 (18.25%)	16	**1.47 × 10**^**−3**^
cGVHD −		14 (21.54%)	0 (0%)	51 (78.46%)	65	
rs10405821	**PALM**					
cGVHD +		0 (0%)	4 (25.00%)	12 (75%)	16	**5.85 × 10**^**−3**^
cGVHD −		14 (21.54%)	3 (4.62%)	48 (73.85%)	65	

^**a**^Lead single nucleotide polymorphisms (SNPs) identified by the Functional Mapping and Annotation of Genome-Wide Association Studies (FUMA) platform SNP2GENE and Multi-marker Analysis of GenoMic Anotation (MAGMA) online tools as significantly associated with the chronic graft-versus-host disease (cGVHD +) group (*p* ≤ 1 × 10^−5^)

^**b**^Gene name corresponding to significant SNP

^**c**^Number of patients presenting with the genotype for the heterozygous allele (one reference and one alternate allele) and the percentage (%) of patients where the genotype was present versus the total number of patients in each group

^**d**^Number of patients presenting with the genotype for the homozygous alternate allele (both alternate alleles) and the percentage (%) of patients where the genotype was present versus the total number of patients in each group

^**e**^Number of patients where no variant/allele was detected and the percentage (%) of patients where no variant (the homozygous reference) was detected versus the total number of patients in each group

^**f**^The total number of patients diagnosed as having cGVHD (cGVHD +) or not (cGVHD −)

^**g**^The Fisher’s exact *p*-value where genotype proportions of heterozygous and homozygous alternate were tested (*α* = 0.05). Significant SNP genotypes are shown in bold

**Fig. 2:**
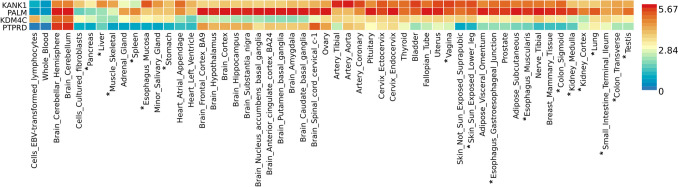
Lead SNP genotype-tissue expression. Legend: Genotype-tissue expression (GTEx_v8_) heatmap rendered using the Functional Mapping and Annotation of Genome-Wide Association Studies (FUMA) online platform’s GENE2FUNC tool. The *y*-axis shows significant genes determined from “lead SNPs” associated with chronic graft-versus-host disease (cGVHD) from a cohort of 81 allogeneic hematopoietic stem cell transplant patients (cGVHD − , *N* = 65; cGVHD + , *N* = 16). The *x*-axis depicts the GTEx_v8_ of 54 tissue types. The level of gene expression per tissue type is shown on a scale of under expressed (blue) to overexpressed (red). cGVHD tissue involvement is depicted with an asterisk (*)

A heatmap of the four lead SNPs tissue expression is shown in Fig. 2. The four genes are functionally expressed in several tissues relevant to cGVHD pathology such as the skin, esophagus, small intestine, and colon. GTEx_v8_ analysis across 54 tissue types showed *PTPRD* as expressed at low levels in 34 tissue types including 16 known to be affected by cGVHD (Fig. 2). *KANK1* was overrepresented in 49 of 54 tissue types, 14 of which have cGVHD involvement (Fig. 2). We determined *KDM4C* was expressed at low levels in five tissue types involved in cGVHD including the pancreas, liver, skeletal muscle, and kidneys. Additionally, our data showed *PALM* being highly expressed in 48 of the GTEx_v8_ 54 tissue types including the lungs and 12 other cGVHD involved tissue types (Fig. 2).

Analysis performed with GENE2FUNC on the four lead SNPs resulted in one significant positional gene set (chr9p24 location) and two curated gene sets (“Davicioni molecular alveolar rhabdomyosarcoma (ARMS) vs. the embryonic form (ERMS) up” and “Snijders amplified in head and neck tumors”). The positional gene set (*n* = 104) contained 3 genes with significant SNPs identified by logistic regression. In addition, the genes *KANK1* and *KDM4C* with the lead SNPs determined by SNP2GENE analysis (padj = 7.20 × 10^−3^), located on chromosome 9, were also members of this positional gene set as shown in Table S1 (Online Resource 5). The curated gene set “Davicioni molecular ARMS vs. ERMS up” (*n* = 20 significant genes of 339 genes) contained three of four lead SNPs representing *KANK1*, *KDM4C*, and *PTPRD* genes per SNP2GENE analysis (padj = 3.59 × 10^−2^) while the “Snijders amplified in head and neck tumors” gene set (*n* = 2 significant genes of 37 genes) contained *KDM4C* and *PTPRD* genes (padj = 3.59 × 10^−2^) (Online Resource 5).

**Fig. 3:**
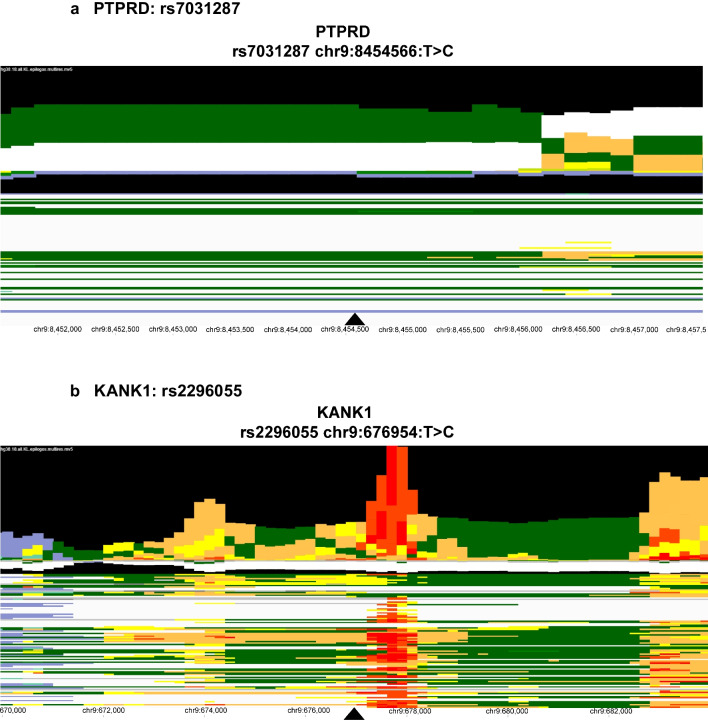

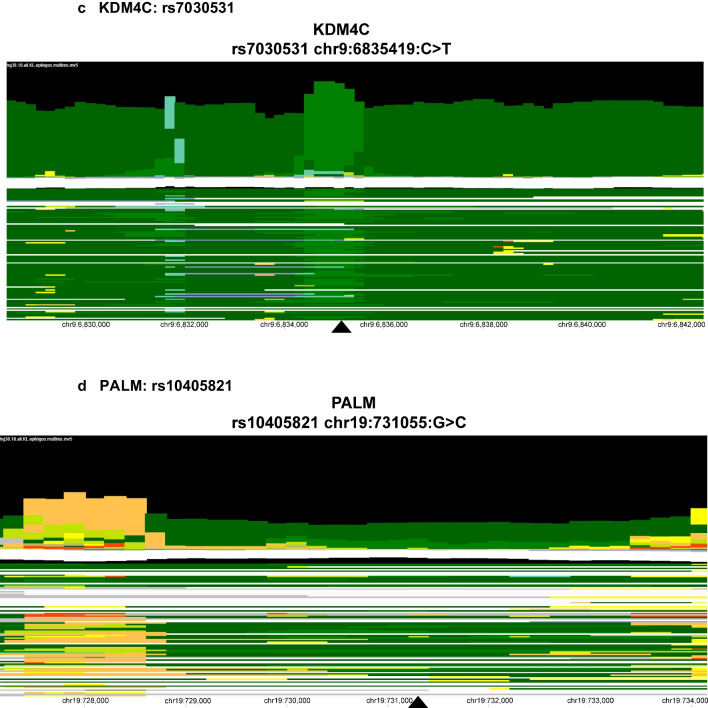
Epilogos of MAGMA identified lead SNPs. Legend: Chromatin state visualization of the lead candidate SNPs (rs7031287 **a**, rs2296055 **b**, rs7030531 **c**, and rs10405821 **d**) determined by Multi-marker Analysis of GenoMic Annotation (MAGMA) using the Epilogos online tool with “hg38” as the human reference genome and “All 127 Roadmap Epigenomes in the 18-state model. The colors represent the following chromatin states: gray  : repressed PolyComb, light purple  : Heterochromatin, pale yellow  : active enhancer, yellow  : weak enhancer, lime green  : geneic enhancer, green  : strong transcription, dark green  : weak transcription, orange  : flanking TSS upstream, red  : active TSS, blue  : ZNF genes and repeats

The SNP in *PTPRD* rs7031387 (T > C) (Fig. 3) is an intron variant within an area of weak transcription in the epigenome and has a global ALFA (allele frequency aggregator) alternate allele frequency of 39%. The *KANK1* gene SNP rs2296055 (T > C) is a noncoding transcription variant flanking upstream of an active transcription start site and transcription regions in the epigenome map. The ALFA alternate frequency among the general population is approximately 30% for this variant. The SNP rs7030531 (C > T) is an intron variant residing within a region of strong transcription with zinc finger genes and repeats. This SNP has an ALFA alternate frequency of 31% among the general population. The *PALM* SNP rs10405821, located on chromosome 19, is also an intronic SNP with an alternate allele frequency of 36% and falls downstream of an active enhancer region within a strong transcription portion of the epigenome map (Fig. 3). Visualization of the chromatin state of each lead SNP’s position of the four genes is presented in Fig. 3.

## Discussion

This is the first study to complete a multi-marker gene-set analysis using whole exome sequencing in a cohort of allogeneic HCT patients with cGVHD. The first three SNPs were all located on chromosome 9, the fourth on chromosome 19, all belonging to specific gene sets with biological relevance.

We identified SNP rs7301287 (chr9:8,454,566:T > C) within the *PTPRD* gene that has been previously identified as a variant in a study investigating susceptibility to large-vessel ischemic stroke [34]. Notably, after stroke there is an acute inflammatory response with an influx of neutrophils, T cells, monocytes, and leukocytes within the brain [35, 36]. SNPs within *PTPRD* may also be associated with hematological cancers or blood disorders in general [37–39]. The SNP we identified within *PTPRD* had significantly more homozygous alternate alleles in the cGVHD( +) group than in the cGVHD( −) group, suggesting the homozygous genotype to constitute a risk for cGVHD. *PTPRD* has been classified as a predictive biomarker of immune checkpoint inhibitors in multiple cancer types including non-small cell lung cancer, skin cutaneous melanoma, and gastric cancer [40]. In this study, they found tumor mutational burden and microsatellite instability to be positively correlated with *PTPRD* mutations in multiple cancer types including non-small cell lung cancer, esophagogastric cancer, and colorectal cancer [40]. In head and neck squamous cell carcinoma, *PTPRD* is a known tumor suppressor and mutations lead to increased expression of signal transducer and activator of transcription 3 (*STAT3*) [41]. When *STAT3* is overexpressed in immune cells, it leads to compromised immunity by inhibiting innate and adaptive immune responses, a characteristic of cGVHD [42]. It remains to be confirmed whether a change in *PTPRD* protein expression or the expression of a modified form contributes to cGVHD by upregulating *STAT3*.

The significant SNP identified within *KANK1*, rs2296055 (chr9:676,954:T > C), was previously identified in a study considering variants associated with coronary artery disease in an Indian population of 153 individuals [43]. Interestingly, atherosclerosis is considered primarily a chronic inflammatory condition that may be influenced by leukocyte count, interferon gamma (IFN- γ), and cytokines [44]. Additionally, another study showed that in certain cancers such as ALL *KANK1* is completely deleted [45]. Although not significant, our results suggest the homozygous alternate allele (C/C) within rs2296055 may constitute a risk for cGVHD, while the heterozygous alternate allele (T/C) may be protective. Furthermore, in a study by Medves et al., the authors were able to identify a fusion of *KANK1* and platelet derived growth factor receptor beta (*PDGFRB*) in a patient with a hematological malignancy [46]. The authors suggested that the *KANK1-PDGFRB* hybrid can transform hematopoietic cells and showed that *STAT3* was phosphorylated in the presence of this hybrid. STAT3 can be phosphorylated by receptor-associated Janus kinases (*JAK*), the inhibitors of which are under investigation in clinical trials for cGVHD. Moreover, *KANK1* has been discovered to fuse with neurotrophic receptor tyrosine kinase 3 (*NTRK3*) gene in patient samples with pathologically confirmed metanephric adenoma leading to a subset of B-Raf proto-oncogene, serine/threonine kinase (*BRAF*) mutations [47]. A study by Cui et al. demonstrated that *KANK1* can inhibit cell growth by inducing apoptosis in malignant peripheral nerve sheath tumor cell lines [48]. The gene *KANK1* also functions as a cytoskeleton regulator and has been suggested as having a role in tissue development [45]. Moreover, *KANK1* has been shown to regulate the polymerization of actin by inhibiting RhoA activity, similar to the Rho family of GTPases [45], which include Rho GTPase activating protein 29 (*ARHGAP29*) involved in the maintenance of oral mucosal epithelium integrity [49, 50]. It is therefore conceivable that *KANK1* homozygous mutations in its regulatory sequence could be responsible for impaired tissue reformation and fibrosis characteristic of the third phase of cGVHD. In addition, since *KANK1* function seems to be linked to the *JAK* pathway and has been shown to inhibit IFN-γ to promote T cell apoptosis further investigation to determine its validity as a drug target candidate for the treatment of cGVHD is warranted [51].

The third lead SNP residing on chromosome 9 (rs7030531; chr9:6,835,419:C > T) corresponded to the gene *KDM4C*. No previous studies have associated rs7030531 with disease. This gene has been suggested as possessing oncogenic properties in solid state tumors as well as AML by functioning as a histone H3K9 demethylase [52]. Furthermore, KDM4 demethylases have been determined as integral for long term-maintenance of hematopoiesis using mouse models [53]. It has been suggested that *KDM4C* is essential to the survival of cells under stress and plays a role in kidney development by regulating autophagy [54]. Although it is rare, cases of cGVHD of the kidneys have been reported in approximately 1% of patients undergoing allogeneic HCT and the risk of chronic kidney disease is increased in long-term survivors [55, 56]. While KDM4C was shown to activate HIF1α/VEGFA signaling through the costimulatory factor STAT3, its role in cGVHD remains unclear [57].

Two genes (*KANK1* and *KDM4C*) were a part of the only significant positional gene set (chr9p24) determined. The results suggest that for the positional gene set located on chromosome 9, the chromatin state of the relevant regions might be significantly impacted by the SNPs. Chromosome 9 houses the largest cluster of interferons in the genome and contains genes related to cell growth and regulation as well as tumor suppressors [58–60]. In a previous study, ABL proto-oncogene 1, non-receptor tyrosine kinase (*ABL1*) located on chromosome 9 was found to fuse with multiple genes which are associated with leukemia [61]. Additionally, this locus is related to diseases including but not limited to renal cell carcinoma, hepatocellular carcinoma, pancreatic carcinoma, ALL, breast cancer, obsessive–compulsive disorder, and monosomy 9p syndrome [45]. Considering chromosome 9 has been associated with leukemias and contains many interferons which are an integral part of the cGVHD pathogenesis, this positional gene set should be further explored for its role in cGVHD.

A study investigating genetic variations affecting gene expression using the Avon Longitudinal Study of Parents and Children cohort identified rs10405821 meaning this SNP could potentially affect trans gene expression [62]. It is worth mentioning, dysregulation of gene expression is associated with inflammatory pathways such as monocyte cytokine responses after lipopolysaccharide stimulation that activate toll-like receptors in children with autism disorders [63]. The SNP rs10405821 is within the gene *PALM,* encoding paralemmin-1 a membrane anchored cytoplasmic facing palmitoylated protein [64]. Paralemmin-1 is abundant in brain tissue but is also well expressed in cGVHD relevant tissues such as lung (Fig. 2) [64]. Paralemmin-1 is a dynamic regulator of intracellular vesicle trafficking, cell shape, and movement [39]. A study by Turk et al. found that *PALM* was upregulated in cell lines representative of estrogen-receptor positive breast cancers [65]. Another member of the PALM family of proteins, *PALM3*, was previously shown to be upregulated by lipopolysaccharide-stimulation in alveolar epithelial cell lines and to be involved in lipopolysaccharide-toll-like receptor-4 signaling in alveolar macrophages. Meanwhile, downregulation of *PALM3* after lipopolysaccharide-stimulation can improve the severity of lung injury and can inhibit pro-inflammatory cytokines in human lung adenocarcinoma cells [65]. It has been suggested that lipopolysaccharide stimulation through bacterial metabolites may induce differentiation of toll-like receptor 4 which could lead to GVHD [66]. The lungs can be significantly affected in cGVHD, considering that cGVHD is considered “severe” if the patient is classified with a lung score of two or more. Whether dysregulation of *PALM* expression has an impact on cGVHD severity through lung injury remains a matter of investigation. This gene was the only gene with significant SNPs on chromosome 19. Chromosome 19 contains a gene region of immunoglobulin like domains that can recognize polymorphic epitopes of HLA class one molecules [67]. In patients with AML undergoing HCT, these domains have been reported to influence treatment outcomes and risk of GVHD [68]. Additionally, abnormalities in chromosome 19 have been associated with megakaryoblastic leukemias and AML [69, 70]. Interestingly, since SNPs were found in three genes (i.e., *PTPRD*, *KANK1*, *KDM4C*) involved in the STAT3 signaling pathway, one possible functional relationship with paralemmin-1 is that STAT3 may be palmitoylated and co-localize with intracellular vesicles which transit to the perinuclear membrane [71, 72]. More research is needed (i) to determine whether ineffectiveness of JAK inhibitors in treating cGVHD relates to such alternative path of translocation of the unphosphorylated form of STAT3 to the nucleus and (ii) to confirm the involvement of *PALM* polymorphisms in cGVHD risk and patient outcomes.

Davicioni molecular ARMS vs. molecular ERMS is a human gene set where genes are upregulated in ARMS compared to the ERMS class of tumors [73]. ARMS and ERMS tumors mainly affect children and young adults. ARMS tumors normally affect muscles in the legs, arms, or trunk, while ERMS affect muscles of the head, neck, or genitourinary tract [74]. The second curated gene set was “Snijders amplified in head and neck cancer tumors” where deregulation of genes in this gene set relate to developmental and differentiation pathways as well as cell misspecification in oral squamous cell carcinoma [75]. How similar biological disturbances related to these gene sets impact the development of hematological cancers or blood disorders as well as the incidence and severity of cGVHD remain unknown. Furthermore, all four lead SNPs may have an impact on the transcriptional regulation of their respective associated gene, most likely as a gain in function by stimulating STAT3 pathway. Finally, among genes involved in cGVHD susceptibility identified in previous SNP association studies it is notable that CCR6 can induce phosphorylation of STAT3 in asthma and that STAT3 is an important binding partner of FGDR1 in the development of non-small cell lung cancer [13, 76, 77].

## Limitations

Although our results provide possible genomic risk loci and insights into the development of cGVHD, the sample size is a limiting factor of this analysis. Indeed, of the 16 cGVHD + patients, 9 had oral signs of GVHD (i.e., lichenoid lesion, erythematous lesion, ulceration, and swollen minor salivary glands) which would not achieve sufficient power to identify associated SNPs. Additionally, the absence of clinical data on the site of cGVHD development and ethnicity of patients due to being a multi-national study limits the scope of this study. Collection of site and ethnicity data for cGVHD along with a larger cohort of allogeneic HCT patients presenting with cGVHD are needed to better investigate susceptibility to cGVHD and to rule out significant findings being only due to other genetic factors or cancer type. Another limitation is the lower-than-expected rate of cGHVD occurrence in the present study, which might correspond to a selection bias. Furthermore, the validation of SNP effects at a functional level experimentally are imperative to identify drug targets to alleviate the long-term effects of cGVHD.

## Conclusions

Our data suggest that there is genetic susceptibility to cGVHD in patients undergoing allogeneic HCT which strongly involves polymorphisms in genes located on chromosome 9, with the possibility of concerted mechanisms of action. Mutations in *PTPRD*, *KANK1*, *KDM4C*, and/or *PALM* may confer predispositions to cGVHD incidence or severity requiring confirmation and validation in future studies to aid in the future drug development for cGVHD.

### Supplementary information

Below is the link to the electronic supplementary material.

Supplementary file1 (CSV 82 KB)

Supplementary file2 (DOCX 2.54 MB)

Supplementary file3 (DOCX 483 KB)

Supplementary file4 (7Z 36732 KB)

Supplementary file5 (DOCX 16.5 KB)

## Data Availability

All data and accompanying scripts are available via the Translational Research Lab Github repository (https://github.com/mbeckm01/GHVD_Exome).
