# Supplementary material for: Identification of single nucleotide polymorphisms (SNPs) associated with chronic graft-versus-host disease in patients undergoing allogeneic hematopoietic cell transplantation
Source: Support Care Cancer. 2023 Sep 21;31(10):587. doi: 10.1007/s00520-023-08044-3 (PMC10511391; doi:10.1007/s00520-023-08044-3)
Supplement: Supplementary file 2 — Supplementary file2 (DOCX 2.54 MB) [file 520_2023_8044_MOESM2_ESM.docx]

**Figure S1. Visualization of SNP profile-based principal component analysis**

**
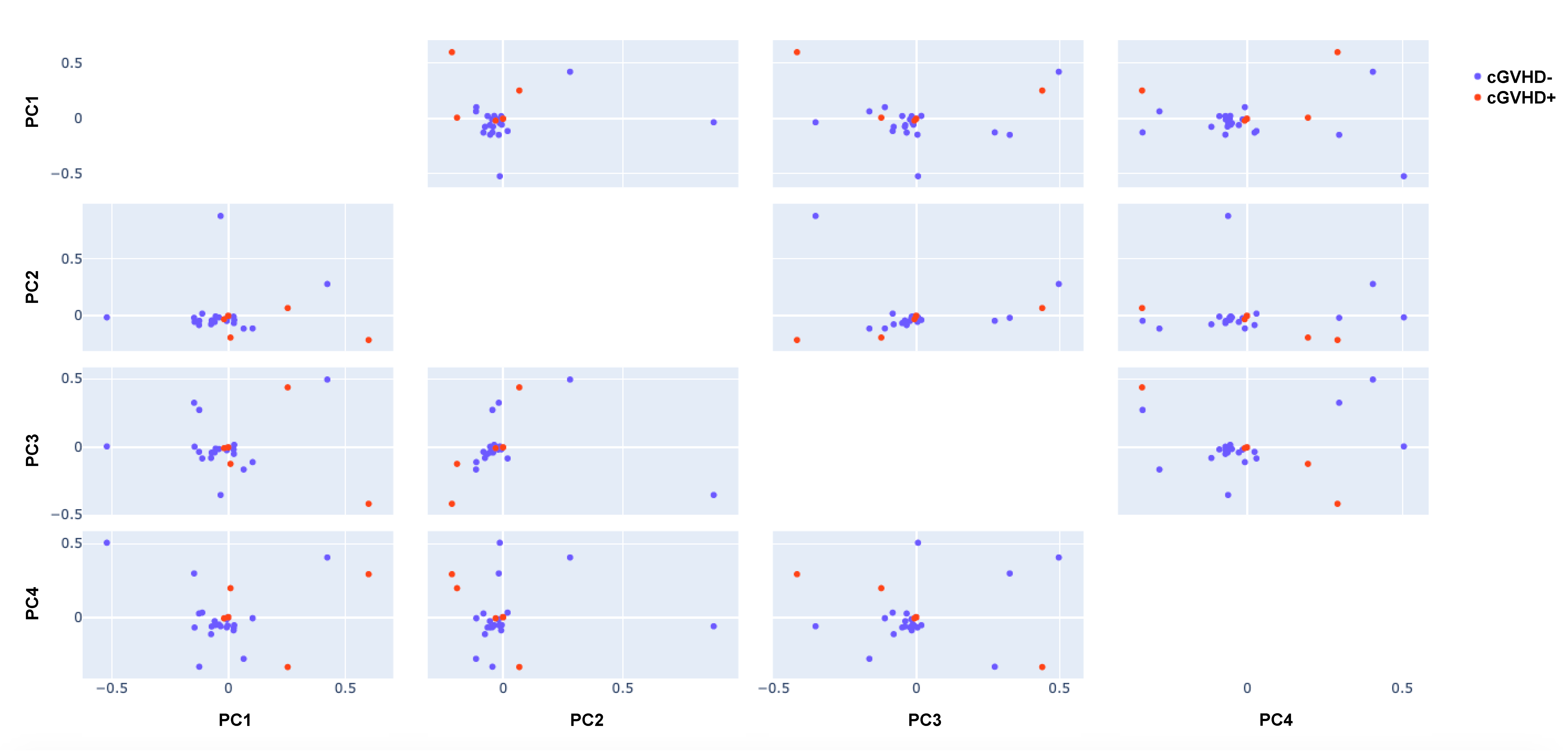
**

**Legend.**

SNP-based visualization of principal components for cGVHD- group (N=66, blue) compared to the cGVHD+ group (N=16; red) from eigenvalues generated using PLINK2_v3.7_ shows no significant differences in the variation of SNP features in each group. Visualization of the first four principal components (out of a total of 10) was created using Python_v3.9.13_, plotly_v5.11.0_ and sklearn_v1.1.1_ opensource python packages.
