# Supplementary material for: Identification of single nucleotide polymorphisms (SNPs) associated with chronic graft-versus-host disease in patients undergoing allogeneic hematopoietic cell transplantation
Source: Support Care Cancer. 2023 Sep 21;31(10):587. doi: 10.1007/s00520-023-08044-3 (PMC10511391; doi:10.1007/s00520-023-08044-3)
Supplement: Supplementary file 3 — Supplementary file3 (DOCX 483 KB) [file 520_2023_8044_MOESM3_ESM.docx]

**Figure S2. Manhattan plots**

1. Genome wide association study summary statistics


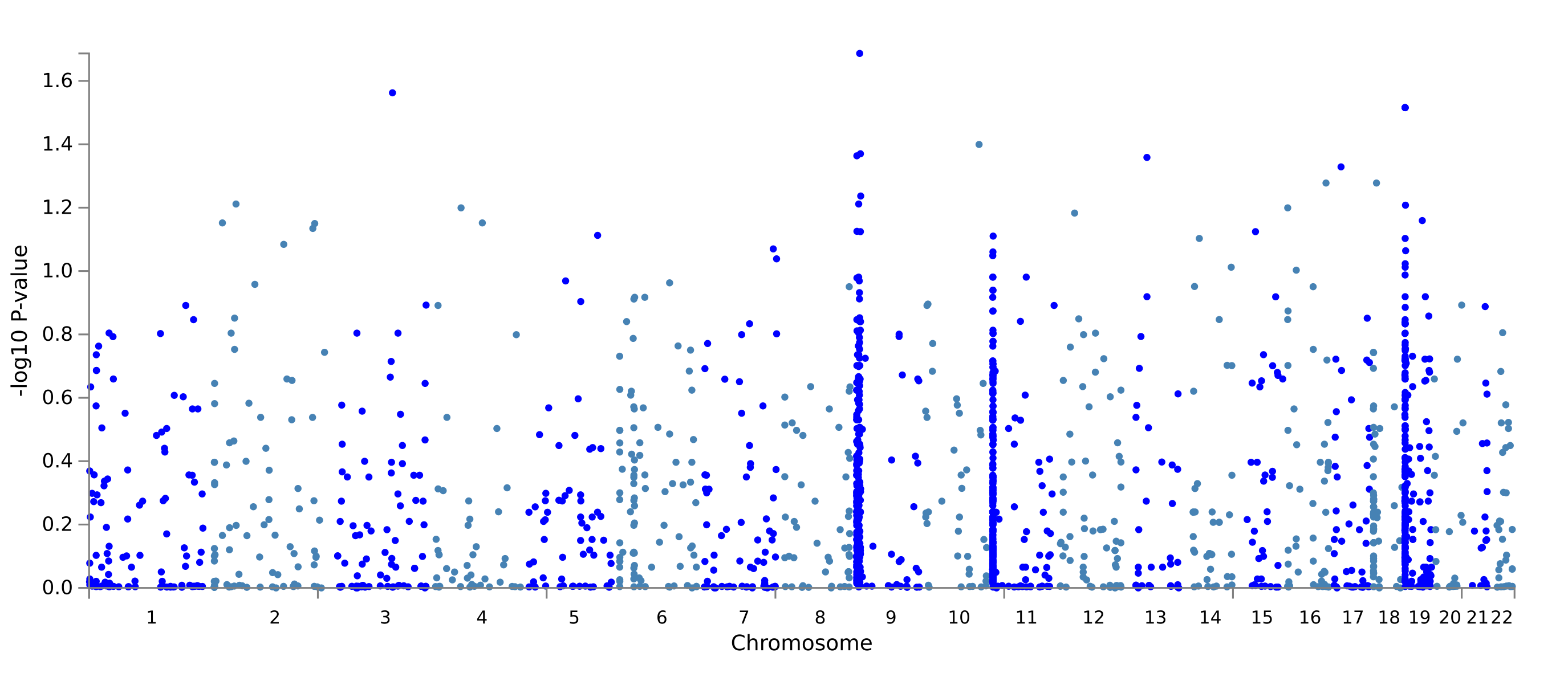


1. Gene-based test


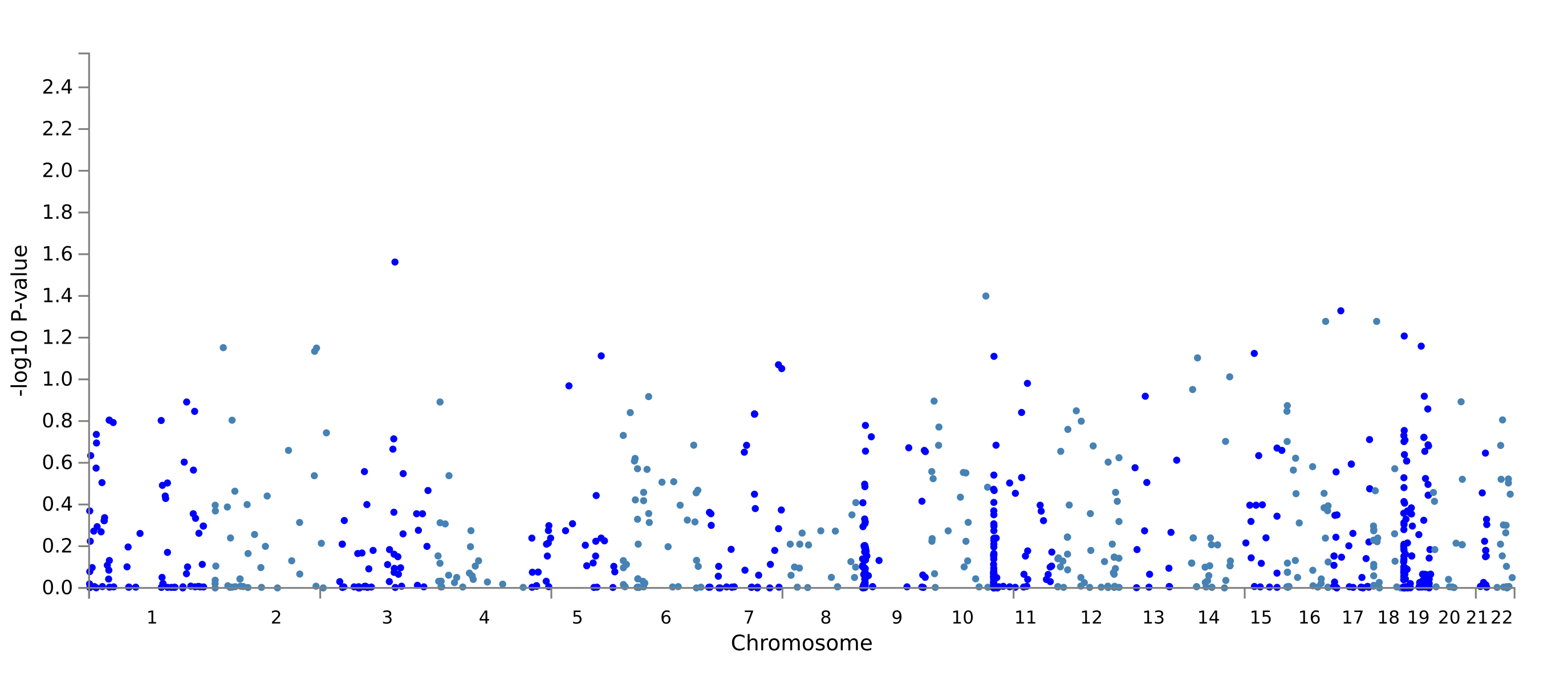


**Legend.**

Manhattan plots for (**a**) genome wide association study (GWAS) summary statistics based on PLINK2_v3.7_ input of significant mapped candidate single nucleotide polymorphisms (SNPs) (n=909 unique out of 986 total) associated with the chronic graft-versus-host-disease positive (cGVHD+) group and (**b**) gene-based test computed by the Multi-marker Analysis of GenoMic Annotation (MAGMA) tool based on GWAS summary statistics. Input SNPs were mapped to 804 protein coding genes. Filtering was completed only for SNPs with a p-value ≤1x10^-5^. Genes were determined as significant at a p-value of 6.219x10^-5^ (0.05/804).
