# Supplementary material for: Identification of single nucleotide polymorphisms (SNPs) associated with chronic graft-versus-host disease in patients undergoing allogeneic hematopoietic cell transplantation
Source: Support Care Cancer. 2023 Sep 21;31(10):587. doi: 10.1007/s00520-023-08044-3 (PMC10511391; doi:10.1007/s00520-023-08044-3)
Supplement: Supplementary file 5 — Supplementary file5 (DOCX 16.5 KB) [file 520_2023_8044_MOESM5_ESM.docx]

**Table S1. Positional and curated gene sets**

**a. Positional gene set**

| **Gene set^a^** | **Entrez gene symbol^b^** | **Overlapping genes^c^** | **Adjusted p-value^d^** |
| --- | --- | --- | --- |
| chr9p24 (n=104) | **ACTG1P14**, AK3, AK4P4, ATP5PDP2, CARM1P1, CBWD1, CD274, CDC37L1, CDC37L1-DT, CSNK1G2P1, DDX11L5, DMAC1, DMRT1, DMRT2, DMRT3, DOCK8, DOCK8-AS1,  DOCK8-AS2, ECM1P1, EIF1P1, ERMP1, FAM138C, FOXD4, GLDC, GLIS3, GLIS3-AS1, GLIS3-AS2, GPS2P1, GTF3AP1, H3P29, HMGN2P31, HNRNPA1P41, IGHEP2, IL33, INSL4, INSL6, JAK2, **KANK1**, KCNV2, **KDM4C**, KIAA2026, KLF4P1, LINC01230, LINC01231, LINC01388, LINC02851, MIR101-2, MIR1302-9, MIR1302-9HG, MIR4665, MLANA, MTATP6P11, MTCO1P11, MTCO2P11, MTCO3P11, MTND1P11, MTND4P14, MTND5P14, MTND6P5, PDCD1LG2, PDSS1P1, PGM5P3-AS1, PLGRKT, PLPP6, PPIAP33, PRELID3BP11, PTPRD-AS1, PUM3, RANBP6, RCL1, RFX3, RFX3-DT, RIC1, RLN1, RLN2, RN7SL123P, RN7SL25P, RN7SL592P, RNA5SP279, RNF152P1, RNF2P1, RNU2-25P, RNU6-1073P, RNU6-1327P, RNU6-694P, RNU7-185P, RPL12P25, RPL23AP57, RPL35AP20, RPL4P5, RPS27AP14, RPS3AP54, SELENOTP1, SLC1A1, SMARCA2, SMARCA2-AS1, SNRPEP2, SPATA6L, TCF3P1, TPD52L3, UHRF2, VLDLR,VLDLR-AS1, WASHC1 | **KANK1, KDM4C** | 7.20x10^-3^ |

**b. Curated gene sets**

| **Gene set** | **Entrez gene symbol** | **Overlapping genes** | **Adjusted p-value** |
| --- | --- | --- | --- |
| Davicioni molecular ARMS vs. ERMS up  (n=339) | ABAT, ABCG1, **ACO1**, ACOT7, ACOT9, ACYP1, ADAM10, ADK, ADRA2A, ADRA2C, AGTPBP1, AKT3, AKTIP, **ALK**, AMZ2, ANK2, ANKRD17, **ANKS1A**, AP5M1, **ARHGAP25**, ARHGAP26, ARHGAP6, ARHGEF2, ARHGEF4, ARL3, ARPP19, ARRB1, ASCC3, ASRGL1, ASS1, ASTN2, B3GAT1, BBIP1, BCL11A, BCL2L13, BLCAP, BMERB1, BMP5, BRD3, BRINP1, C14orf132, CAAP1, CAMSAP1, CAPN6, CCNH, CCP110, CD47, CDC14B, CDC42EP3, CDH3, CDK14, CDK2AP1, CDS2, **CDYL**, CELA2B, CEP104, CEP68, CHD7, CHRNB3, CHST8, CLASP2, CLCN3, CLCN5, CLIP1, CNR1, COBL, COG2, COL18A1, COQ9, COX7A1, CPD, CRIP2, CRMP1, CSRNP2, CTBP2, CUL3, CYB561, DAG1, DAPK1, DCX, DDR1, DET1, DHFR, DIO2, DIPK1A, DISC1, DIXDC1, DLGAP2, DLK2, DNASE1L1, DNMBP, DOCK9, DST, DTWD1, DZIP3, ECHS1, EDN3, EFHD2, EIF1AX, ELMO1, ELOVL2, EMC9, ENDOG, ENO3, ERI2, ERP44, **EYA2**, FAN1, FBXL5, FBXO9, FGFR2, FGFR4, FLVCR2, FOXF1, FOXO1, FRMPD1, FRY, FRYL, FTO, GABPB1-IT1, GADD45A, GADD45G, GCA, GGNBP2, GMIP, GNB1, GNE, GOSR1, GOT2, GREM1, GYPC, HCCS, HDAC5, HERC2, HMGN4, HPRT1, HSBP1, HSF2, IFT81, IL4R, IQCG, ISCA1, JAKMIP2, JARID2, **KANK1**, KATNIP, KCND3, KCNN3, **KDM4C**, KIF1B, KLF7, LINC00588, LIPG, LMO4, LPAR2, LRRFIP2, LSP1P5, MAGI2, MAMLD1, MAN1C1, MAP1LC3B, MAPK6, MARCHF3, MARCHF6, MDC1, MED13L, MEGF9, MEOX1, MET, MICAL1, MINDY2, MLH3, MMUT, **MNAT1**, MORC4, MREG, MSRB1, MTMR4, MXRA7, MYB, MYCN, MYLIP, MYMX, MYO18A, MYO1E, MYOD1, MYOG, **NCOA1**, NELL1, NET1, NF1, NHLH1, NKAIN1, NLGN4X, NMRK1, NNT, NOTCH1, NPC1, NPEPPS, NR0B1, NRCAM, NRN1, NRTN, NTRK3, NUDT11, OAT, OLFML2B, OLIG2, PAFAH1B1, PAIP1, PARP1, PAX2, PAX5, PBK, PCMT1, PCNT, PCSK6, PDZRN3, PEBP1, PEG3, PGBD5, PIAS1, PIGN, PIMREG, PIPOX, PITRM1, PKP4, **PLPP1**, PLPPR1, PODXL, POLG2, POPDC3, POR, POU4F1, PPM1H, PPP3CA, PRKAR2B, PRKX, PRMT2, **PRRC2B**, PSEN2, PSMB2, PSME1, PTBP2, **PTPRD**, **PTPRF**, QDPR, RAB28, RANGRF, RAP1GAP2, RAPGEF4, RASSF4, RBM8A, RBPJ, RGS17, **RIPOR2**, RNF11, RNF111, RNF38, RNF5, **RNGTT**, ROGDI, RRAGA, RRAGD, RYR3, S1PR1, SAMD4A, SATB2, SCAMP1, SEL1L3, SEMA5A, SESN1, SGMS1, SGPL1, SH3GLB1, **SHOC2**, **SLC24A3**, SLC27A6, SLC30A9, SLC38A1, SLC46A3, SLC9A6, SMARCA5, SMPDL3A, SNAI1, SOS2, SPATS2L, SPIN2A, SPINT2, SRSF4, STC2, STX7, SUN2, SYN2, SYNCRIP, SYNE2, TAGLN3, TBC1D9, TBCA, TBCD, TEFM, TEX14, TEX2, TFAP2A, TFAP2B, TFF3, THUMPD1, TLK2, TMEM120B, TMEM131L, TMEM260, TMEM47, TNFAIP1, TOM1L1, TOPORS, TOX3, TRIM36, TRIM37, TRPV1, TSC22D2, TSPAN3, TSPYL4, TSTD2, TTC19, TULP4, TXNIP, UBR2, USP12, VIPR2, **WARS1**, WASHC3, WSCD1, WWOX, ZBTB18, ZCCHC14, ZNF248, ZNF330, ZNF43, ZNF91 | **KANK1, KDM4C, PTPRD** | 3.59x10^-2^ |
| Snijders amplified in head and neck tumors  (n=37) | ADAM9, BAG4, BIRC2, BIRC3, CCND1, CD44, CDK6, CTTN, EGFR, EYS, FGF3, FGFR1, FJX1, FZD1, GLI2, IL7R, JAG1, **KDM4C**, LAMA3, MAPK8IP1, MDM2, MMP7, PAK1, PHF21A, PTP4A1, **PTPRD**, PTPRR, RAD1, RBPJ, SKP2, STIM2, TACC1, TLN1, TM4SF1, TRAF6, UHRF2, YAP1 | **KDM4C, PTPRD** | 3.59x10^-2^ |

**^a^**Significant **a.** positional and **b.** curated gene sets determined by GENE2FUNC analysis

**^b^**Entrez gene symbol of genes in the gene set

**^c^**Genes overlapping in GENE2FUNC enrichment analysis

**^d^**Adjusted p-value of the hypergeometric test

Note: Genes with PLINK2_v3.7_ significant SNPs are shown in bold.
